# Mutations in TGF-beta signaling pathway components regulate the *Drosophila melanogaster* lifespan

**DOI:** 10.17912/micropub.biology.001333

**Published:** 2024-11-19

**Authors:** Virginia Fife, Margarita Kyza-Karavioti, Ioannis Eleftherianos

**Affiliations:** 1 Biological Sciences, George Washington University, Washington, Washington, D.C., United States

## Abstract

The evolutionary conserved transforming growth factor beta (TGF-β) signaling pathway participates in the regulation of several cellular functions and tissue homeostasis. In the model *Drosophila melanogaster* , the two TGF-β signaling pathway branches Bone Morphogenic Protein (BMP) and Activin are involved in important developmental and immune processes. Here we examine the effect of mutations in various BMP and Activin signaling molecules on the fly lifespan. We find that loss-of-function fly mutants for distinct Activin and BMP components differentially modulate the fly lifespan. These results indicate that the TGF-β signaling pathways act as regulators of lifespan in the adult *D. melanogaster* .

**Figure 1. Lifespan of Drosophila melanogaster TGF-beta signaling (Activin and BMP branches) pathway mutant adult female and male flies:**
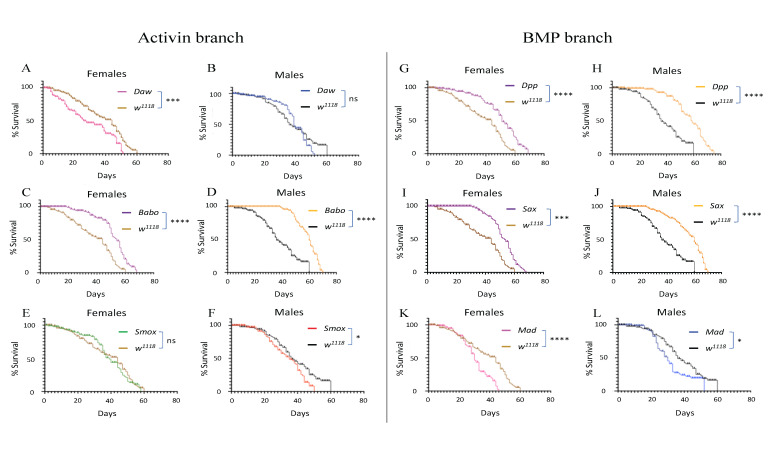
Percent survival of *D. melanogaster* (A) *Daw* female, (B) *Daw* male, (C) *Babo* female, (D) *Babo* male, (E) *Smox* female, (F) *Smox* male, (G) *Dpp* female, (H) *Dpp* male, (I) *Sax* female, (J) *Sax* male, (K) *Mad* female, (L) *Mad* male adult flies. Lifespan experiments were replicated three times and each experiment involved at least 100 females and 100 males from each mutant line and the background controls (****P<0.0001, ***P<0.001, *P<0.05, ns = non-significant).

## Description

In the fruit fly model *Drosophila melanogaster* , the conserved TGF-beta signaling pathway consists of the Bone Morphogenic Protein (BMP) branch and the Activin branch (Peterson and O'Connor, 2014). Each TGF-beta signaling branch involves extracellular ligands, Type I and Type II transmembrane receptors, and intracellular signal transducers. Here, we focused on the Activin branch signaling components Dawdle (Daw, ligand), Baboon (Babo, Type I receptor) and Smad on X (Smox, transducer), and the BMP branch signaling components Decapentaplegic (Dpp, ligand), Saxophone (Sax, Type I receptor) and Mothers against decapentaplegic (Mad, transducer) (Li et al., 2006) .

Previously, we have shown that the TGF-β signaling plays a major role in the anti-nematode immune and metabolic response of *D. melanogaster* (Ozakman and Eleftherianos, 2019; Bastin and Eleftherianos, 2023) . Our recent findings suggest that the TGF-β ligands Daw or Dpp act as regulators of the resistance of *D. melanogaster* adults against infection with two entomopathogenic nematode species, *Heterorhabditis bacteriophora* and *H. gerrardi* , which carry the symbiotic bacteria *Photorhabdus luminescens* and *P. asymbiotica* (Eleftherianos et al., 2016) . Also, expression of the intracellular transducer Mad and the ligand Dpp controls the antimicrobial peptide gene expression after nematode infection, but not after infection with their cognate bacterial symbionts (Patrnogic et al., 2018a). Mad is also activated following expression of the extracellular ligand Scw and the receptors Sax and Babo when *D. melanogaster* adult flies are infected with *H. gerrardi* nematodes (Patrnogic et al., 2018b). Interestingly, TGF-β signaling pathway activity interacts with NF-κB immune signaling activity takes place extracellularly in response to *H. gerrardi* nematode infection of adult flies (Patrnogic et al., 2019) .

In the current work, we investigated the relationship between TGF-β signaling activity and lifespan in *D. melanogaster* . For this, we explored the lifespan of female and male adult flies with loss-of-function mutations in genes coding for various components in the BMP and Activin branches of the TGF-β signaling pathway.

For the Activin branch mutants, we found that *Daw* female mutants had significantly shorter lifespan compared to *w ^1118^* controls (Mantel-Cox, *df* = 1, *P* <0.001) and there were no statistically significant differences between *Daw* male mutants and *w ^1118^* individuals (Figures 1A and 1B). Also, we found that *Babo* female and male mutants had significantly longer lifespan compared to *w ^1118^* controls (Mantel-Cox, *df* = 1, *P* <0.0001) (Figures 1C and 1D). Finally, we showed that although *Smox* female mutants and *w ^1118^* flies lived for approximately 60 days ( Figure 1E ), *Smox* male mutants had significantly shorter lifespan compared to their control counterparts (Mantel-Cox, *df* = 1, *P* <0.05) ( Figure 1F ).

For the BMP branch mutants, we found that both *Dpp* female and male mutants lived significantly longer than *w ^1118^* controls (Mantel-Cox, *df* = 1, *P* <0.0001) (Figures 1G and 1H). A similar pattern was also observed for *Sax* female and male mutant flies which lived significantly longer than *w ^1118^* individuals (Mantel-Cox, *df* = 1, *P* <0.001 and P<0.0001, respectively) (Figures 1I and 1J). Finally, both female and male *Mad* mutants lived significantly shorter than *w ^1118^* background control flies (Mantel-Cox, *df* = 1, *P* <0.0001 and Mantel-Cox, *df* = 1, *P* <0.05, respectively) (Figures 1K and 1L).

A previous study has reported that knocking down Activin signaling in the whole body of adult *D. melanogaster* or specifically in the adult muscle reduces the fly lifespan, and overexpressing the Activin ligands Daw or Myg separately in the muscle of adult flies extends lifespan (Langerak et al., 2018) . This effect was attributed to the requirement of Activin signaling for directing protein homeostasis and directly or indirectly modifying 26S proteasome activity in adult fly muscles. However, an earlier study reported that knockdown of the Activin signaling components Daw, Babo or Smox extends the fly lifespan due to increase in autophagy in muscles (Bai et al., 2013) . Using a different set of *D. melanogaster* TGF-β signaling mutants, here we find that a loss-of-function mutation in *Daw* shortens the lifespan of female flies only, mutation in *Smox* reduces the lifespan of males only, and mutation in Babo extends the lifespan of both sexes. The discrepancy between our observations and those previously obtained is currently unknown and it will be the focus of future studies. Our work further expands previous results by showing that mutating either the Dpp ligand or the Type I receptor Sax of the BMP branch confers a positive effect on the lifespan of female and male flies, and finally mutation in the transducer Mad decreases the lifespan of both sexes. Identifying the functional basis of fly lifespan regulation by interfering with the activity of the Activin and BMP TGF-β signaling branches will be explored in subsequent time.

In conclusion, the current findings reveal the positive and negative effect of TGF-β pathway signaling components on the lifespan of the fruit fly model *D. melanogaster* . Future studies will be performed in the absence or presence of various stresses to dissect the tissue-specific molecular interaction between TGF-β signaling and other pathways such as insulin-like signaling, inactivation of which has been previously shown to extend lifespan in the fly (Hwangbo et al., 2004; Partridge et al., 2011) . This information will contribute towards a better understanding of the regulatory players that operate to maintain homeostasis during aging and the molecular factors that lead to age-dependent senescence of the immune system.

## Methods

**Fly stocks.** The following mutant lines were used in the experiments: P{XP}daw ^d05680^ (stock d05680, The Exelixis Collection, Harvard), w[*]; babo ^32^ /CyO (stock 5399, Bloomington *Drosophila* Stock Center), w ^1118^ ; PBac{Smox-GFP.FLAG}VK00033 (stock 43958, Bloomington *Drosophila* Stock Center), dpp ^s1^ (stock 397, Bloomington *Drosophila* Stock Center), y ^1^ w ^*^ ; sax ^5^ /CyO (stock 8785, Bloomington *Drosophila* Stock Center), PBac{PB}Mad ^c00574^ (stock c00574, The Exelixis Collection, Harvard), w ^1118^ (stock 3605, Bloomington *Drosophila* Stock Center), y ^2^ w ^a^ (stock 189, Bloomington *Drosophila* Stock Center). All fly stocks were reared on Bloomington *Drosophila* Stock Center cornmeal food (LabExpress) supplemented with yeast (Carolina Biological Supply) and maintained in an incubator at 25°C and a 12-hour photoperiod.

**Fly lifespan assessment.** Newly eclosed *D. melanogaster* were collected by anesthetizing the flies under carbon dioxide. They were kept at a density of 10 females and 10 males each per vial. At least 100 females and 100 males were tested for each mutant and wild-type line. Flies were kept on Bloomington *Drosophila* Stock Center cornmeal food (LabExpress) supplemented with yeast (Carolina Biological Supply), and maintained in an incubator at 25 ^o^ C temperature and 12:12-h light:dark photoperiod. All flies were transferred to fresh vials every 3-4 days for the duration of the lifespan experiment. Fly survival was observed and recorded daily during the afternoon.

**Statistical analysis.** GraphPad Prism 9 software was used for statistical analysis of the fly lifespan results. Statistics were performed on data obtained from three independent lifespan experiments. For lifespan curves, pairwise comparisons of each experimental group with its control were carried out using a log-rank (Mantel–Cox) test. All error bars represent standard error of mean.
